# Antibodies to Nucleocapsid Are Not Diagnostic for Long COVID

**DOI:** 10.1128/spectrum.04900-22

**Published:** 2023-02-21

**Authors:** C. T. Brandt, L. Wiese, K. M. Christiansen, J. Agergaard

**Affiliations:** a Department of Infectious Diseases, Zealand University Hospital, University of Copenhagen, Roskilde, Denmark; b Department of Infectious Diseases, Odense University Hospital, Odense, Denmark; c Department of Infectious Diseases, Aarhus University Hospital, Aarhus, Denmark; Thomas Jefferson University

## LETTER

Studying the long-term effects of COVID-19 (long COVID) among members of a Facebook group dedicated to individuals who suspect they may have sequelae from COVID-19 is innovative (1).

The study by Fogh et al. emphasizes the importance of thorough investigation of patients with symptoms resembling long COVID to rule out other causes of malaise. Fogh et al. used antibodies against the nucleocapsid antigen as a proxy for detecting previous COVID infection among Danish individuals with a perception that they suffer from long COVID. The finding that 32% of the participants did not have detectable antinucleocapsid antibodies leads to the conclusion that their symptoms were most likely not related to COVID-19. The low number of respondents—below 5%—is likely to have introduced a bias of uncertain significance besides skewed gender distribution.

The acquired data do, however, only to a limited extent support the study conclusions.

The study premises where Facebook group members with negative screening to SARS-CoV-2 nucleocapsid protein are considered not to have had COVID is challenging since 30 to 40% of patients will not have persisting antibodies at the time of recruitment (2). Furthermore, patients with mild COVID have an antibody response with an even more rapid decay (2, 3). The results presented by Fogh et al. confirm these results by showing significantly increased time from COVID in seronegative individuals compared to seropositive (317 versus 214 days). Close to 90% of the included group members had COVID PCR tests performed. These results could be included as confirmative data for infection as a more reliable indicator of COVID disease status. All available data are important in order to characterize long COVID, and a positive SARS-CoV-2 test is part of the long COVID diagnosis. It is important to use test-positive patients when we characterize long COVID symptoms and search for new treatment options for long COVID patients.
